# Items

**Published:** 1882-05

**Authors:** 


### Items.

Official List of Changes of Stations and Duties of Medical Officers of the United States Marine Hospital Service, January 1, 1881, to March 31, 1882.

Bailhache, P. H., Surgeon. To proceed to Richmond, Va., as inspector, January 31, 1882.

Vansant, John, Surgeon. Detailed as President Board of Survey, physical examination of officers of the Revenue Marine Service; March 18, 1882.

Wyman, Walter, Surgeon. When relieved by Surgeon Austin to proceed to Baltimore, Md., and assume charge of the service at that port; March 4, 1882.

Fessenden, C. S. D., Surgeon. To proceed to Greenport, Sag Harbor, New York, as inspector, January 26, 1882. Detailed as President Board of Survey for the physical examination of pilot, to meet at Boston, Mass., February 16, 1882; February 7, 1882.

Purviance, George, Surgeon. To proceed to Gloucester, Mass., to extend relief to shipwrecked seamen; January 12, 1882. Detailed as Recorder Board of Survey for the physical examination of pilot, to meet at Boston, Mass., February 16, 1882; February 7, 1882.

Austin, H. W., Surgeon. To proceed to Cincinnati, Ohio, and assume charge of the service at that port, relieving Surgeon Wyman; March 4, 1882.

Godfrey, John, Passed Assistant Surgeon. When relieved by Passed Assistant Surgeon Goldsborough, to proceed to New Orleans, La., and assume charge of the service of that port; March 4, 1882. Granted leave of absence for eight days; March 24, 1882

Irwin, Fairfax, Assistant Surgeon. Granted leave of absence for seven days; January 18, 1882.

O'Connor, F. J., Assistant Surgeon. To report to General Superintendent, L. S. S., for duty as member of board to examine keepers and crews of the Life Saving Service; January 4, 1882. Relieved on account of sickness, and directed to report to Surgeon in-charge, New York, N. Y., for temporary duty; January 18, 1882. To proceed to Detroit, Mich., and report for duty to the Surgeon-in-charge; February 9, 1882.

Banks, C. E., Assistant Surgeon. To proceed to Portland, Oregon, and assume charge of the service at that port; March 1, 1882.

Devan, S. C., Assistant Surgeon. Detailed as Recorder Board of Survey, physical examination of officer of the Revenue Marine Service; March 18, 1882.

Urquhart, F. M., Assistant Surgeon. To report to General Superintendent, L. S. S., for duty as member of board to examine keepers and crews of the Life Saving Service; January 18, 1882.

Kalloch, P. C., Assistant Surgeon. To proceed to New York, N. Y., for temporary duty; January 23, 1882.

RESIGNATION.

Hebersmith, Ernest. Resignation as Surgeon accepted, to take effect November 26, 1881; January 17, 1882.

PROMOTION.

Smith, Henry, Surgeon. Promoted and appointed Surgeon from January 20, 1882; January 20. 1882.

APPOINTMENT.

Kalloch, Parker C., M. D., of Pennsylvania, having passed successfully the examination required by the Regulations, was appointed an Assistant Surgeon by the Secretary of the Treasury, January 23, 1882.

---

PROF. JOHN E. OWENS has resigned the chair of Orthopedic Surgery in Rush Medical College to accept the chair of Surgical Anatomy and Operative Surgery in Chicago Medical College.

THE RADICAL CURE OF CANCER. Announcement Concerning Essays upon this subject offered in competition for prizes.

The undersigned, who in October last, was delegated to receive competing essays on the subject of the radical cure of malignant disease, announces that three essays were presented. In the consideration of their merits the assistance of Dr. George B. Shattuck, Editor of the *Boston Medical and Surgical Journal* was invoked; and it has been decided that no essay is worthy of a prize.

The same subject namely *The Probability of the Discovery of a Cure of Malignant Disease, and the Line of Study or Experimentation likely to bring such a Cure to light*, is proposed for essays to be presented in competition not later than the first day of December, eighteen hundred and eighty-three (1883), to the undersigned, who, with such assistance as he may select, will be the judge of their merits.

For the best essay on the above subject a prize of ONE THOUSAND DOLLARS will be given, the right being reserved to withhold the prize in case no essay of sufficient merit be presented.

The sum above mentioned has already been deposited in the New England Trust Company, of Boston, subject to the call of the judges.

The essays must be legibly written in English, and neatly bound. Each one must bear a motto, and be accompanied by a sealed envleope bearing the same motto, and inclosing the name and address of the writer. They will all remain in the possession of the donor of the prize for convenience of reference, and the privilege is claimed to publish the successful one, with the name of the writer. No writer however, surrenders the privilege of retaining a copy of his essay, and publishing it.

The decision concernig the merits of the essays will be made chifley from a practical stand-point, it being the object of the donor of the prize to obtain suggestions by which a search for a cure for cancer may be instituted.

For the donor,

J. COLLINS WARREN, M.D.,

58 *Beacon Street*, *Boston*, *Mass.*, *U.S.A.*

March, 1882.

PROF. D. W. GRAHAM has been elected Secretary of the Woman's Medical College. Prof. W. J. Maynard has resigned the chair of Therapeutics and accepted that of Dermatology and Venereal Diseases. Prof. Marie J. Mergler has been made Professor of Therapeutics, in addition to the chair of Materia Medica occupied by her.

PROF. F. L. WADSWORTH has resigned the Lectureship of Physiology in the spring course of Rush Medical College.

PROF. I. N. DANFORTH has resigned the Professorship of Pathology in Rush Medical College, and accepted a chair of Clinical Medicine in the Chicago Medical College.

DR. E. C. DUDLEY has accepted the chair of Medical and Surgical Diseases of Women and of Clinical Gynæcology in Chicago Medical College.

DR. WALTER HAY has accepted the chair of Materia Medica in Chicago Medical College.

DR. OSCAR C. DEWOLF has received the chair of State Medicine and Hygiene in Chicago Medical College.

DR. CHRISTIAN FENGER has accepted the chair of Pathology and Surgical Diseases of the Genito-Urinary Organs in Chicago Medical College.

DR. F. C. SCHAEFFER has been appointed Professor of Anatomy in Chicago Medical College.

DR. ROSWELL PARK has received the appointment to the Lectureship of Surgery in Rush Medical College.

DR. FRANK BILLINGS has been appointed Demonstrator of Anatomy in Chicago Medical College.

Dr. M. C. GREEN, of Chicago Medical College, has been made assistant to Department of Nervous Diseases in St. Joseph's Hospital, Chicago.

